# What Are the Physical Demands of Sexual Intercourse? A Systematic Review of the Literature

**DOI:** 10.1007/s10508-021-02246-8

**Published:** 2022-02-11

**Authors:** José M. Oliva-Lozano, Fernando Alacid, Pedro A. López-Miñarro, José M. Muyor

**Affiliations:** 1 Health Research Centre, Faculty of Educational Sciences, University of Almería, Edificio de Humanidades A, Ctra. Sacramento s/n. 04120, La Cañada de San Urbano, Almería, Spain; 2 Department of Physical Education, University of Murcia, Murcia, Spain; 3 Laboratory of Kinesiology, Biomechanics and Ergonomics, Research Central Services, University of Almería, Almería, Spain

**Keywords:** Sexual activity, Coitus, Physical activity, Exercise, Sexual intercourse

## Abstract

The aim of this study was to systematically review the literature investigating the physical demands of sexual intercourse and to synthesize the evidence related to this research topic. Original studies published on PubMed, Scopus, and Web of Science up until April 2020 were examined. The Effective Public Health Practice Project scale was used to assess the methodological quality of each study. Eighteen studies analyzed physical demands during sexual intercourse through the physiological demands (*n* = 14) and kinematics (*n* = 4) of sexual intercourse. Sexual intercourse can elicit an energy expenditure of ~ 100 kcal (or ~ 6 metabolic equivalent units, METs) during the activity, mean heart rates between ~ 90 and ~ 130 beats per minute (bpm), and peak heart rates up to ~ 170 bpm. However, these physical demands may vary depending on health status, intercourse position, activity duration, intercourse phase, and sex differences. The movement pattern was cyclic in all positions and the greatest demands in lumbar spine flexion were found in the missionary positions for women. Missionary and side-lying positions elicited the greatest lumbar flexion movement in men. Regarding the movement of the hip joint during sexual intercourse, flexion, abduction, and external rotation mainly characterized the woman’s movement while external rotation did so in the man. In conclusion, sexual intercourse may elicit moderate intensity physical demands, but these demands vary depending on contextual variables. In addition, not only studies combining physiological and kinematic analyses are necessary but also more high-quality studies need to be published in order to have a better understanding of the physical demands of sexual intercourse.

## Introduction

Sexual activity is an integral part of human life, and one that ensures our species does not go extinct. With it go physical and psychological health benefits when good and safe practices are followed (Levin, 2007). Sexual intercourse involves physical movement that expends energy for both men and women (Liu et al., 2016), and it is thus recognized as a form of physical activity (Ainsworth et al., 1993, 2000, 2011). Currently, there are many health recommendations on physical activity available in the literature, which attempt to deal with sedentary behaviors, obesity, and other health-related issues (Foster et al., 2018; Füzéki & Banzer, 2018; Garber et al., 2011). These recommendations suggest that regular exercise, and a gradual increase in exercise volume and intensity, may decrease the risk of developing health problems (e.g., cardiovascular disease) (Garber et al., 2011). However, no recommendations on volume or intensity have specifically focused on sexual intercourse to date.

Within the sexual intercourse context, the volume could be defined as the workload (e.g., the intercourse duration or the total of penetration cycles) while the intensity would represent the degree of difficulty or physical exertion (e.g., the speed of action, heart rate, and energy demands). Consequently, these variables would comprise the physical demands of sexual intercourse (i.e., the overall level of physical exertion required to perform this activity). The most recent version of the Compendium of Physical Activities categorized sexual activity as having 2.8 metabolic equivalent units (METs) (Ainsworth et al., 2011), which corresponds to low intensity physical activity (Garber et al., 2011; Haskell et al., 2007). However, a more recent study concluded that sexual intercourse intensity could also be moderate or vigorous, and that it differs significantly between men and women (Frappier et al., 2013).

These findings imply that controversy exists within the literature (e.g., the METs required during sexual intercourse), and research is needed in this field of study. Furthermore, previous research concluded that many beliefs persist in our society, given the absence of supporting scientific evidence (Casazza et al., 2013). For example, Casazza et al. considered the belief that a bout of sexual activity could burn between 100 and 300 kcal to be a myth, since these results appear to be unsupported by the standardized energy expenditure formula (kcal/min = estimated MET of the activity × 0.0175 x weight in kilograms) (Casazza et al., 2013; Jetté et al., 1990; Pinheiro Volp et al., 2011).

Regarding the physical demands of sexual intercourse, variables such as intercourse position, health status, or physiological and kinematical demands have been independently studied. In addition, understanding sexual intercourse as a form of physical activity may help us to understand the relationships between health and disease (Ainsworth et al., 1993). Therefore, the aim of this study was to systematically review the literature investigating the physical demands of sexual intercourse and synthesize the evidence related to this research topic.

## Method

### Search Strategy

A systematic review of the literature was conducted on the PubMed, Scopus, and Web of Science electronic databases. The last systematic review was performed on April 4th, 2020. The Preferred Reporting Items for Systematic Reviews and Meta-analyses (PRISMA) checklist (Liberati et al., 2009; Moher et al., 2009) was followed. The protocol for this systematic review was registered on PROSPERO (CRD42020179979). The keywords selected for the search strategy combined words related to the physical demands (“cardiovascular,” “heart rate,” “blood pressure,” “energy,” “kcal,” “physiology,” “physiological,” “metabolic equivalent units,” “MET,” “kinematic*,” “motion,” “cycle,” “range of motion,” “velocity,” “speed,” “acceleration,” “entropy,” “intensity,” “physical load,” “physical demands,” “movement,” or “displacement”) and words related to sexual intercourse (“intercourse,” “coitus,” or “sexual activity”). Specifically, both groups (i.e., physical demands and sexual intercourse) were linked by AND as a Boolean operator.

All search terms under one heading are linked by a Boolean OR, that is, this term or that term, and each group is linked by a Boolean AND, that is, this group of terms and that group of terms.

### Study Selection

Studies were included in this systematic review if they were: (a) cross-sectional or longitudinal (cohort or experimental) studies; (b) in English; and (c) analyzing the physical demands of sexual activity which involved sexual intercourse. Congress or conference proceedings, abstracts, books, and theses were excluded.

**Fig. 1:**
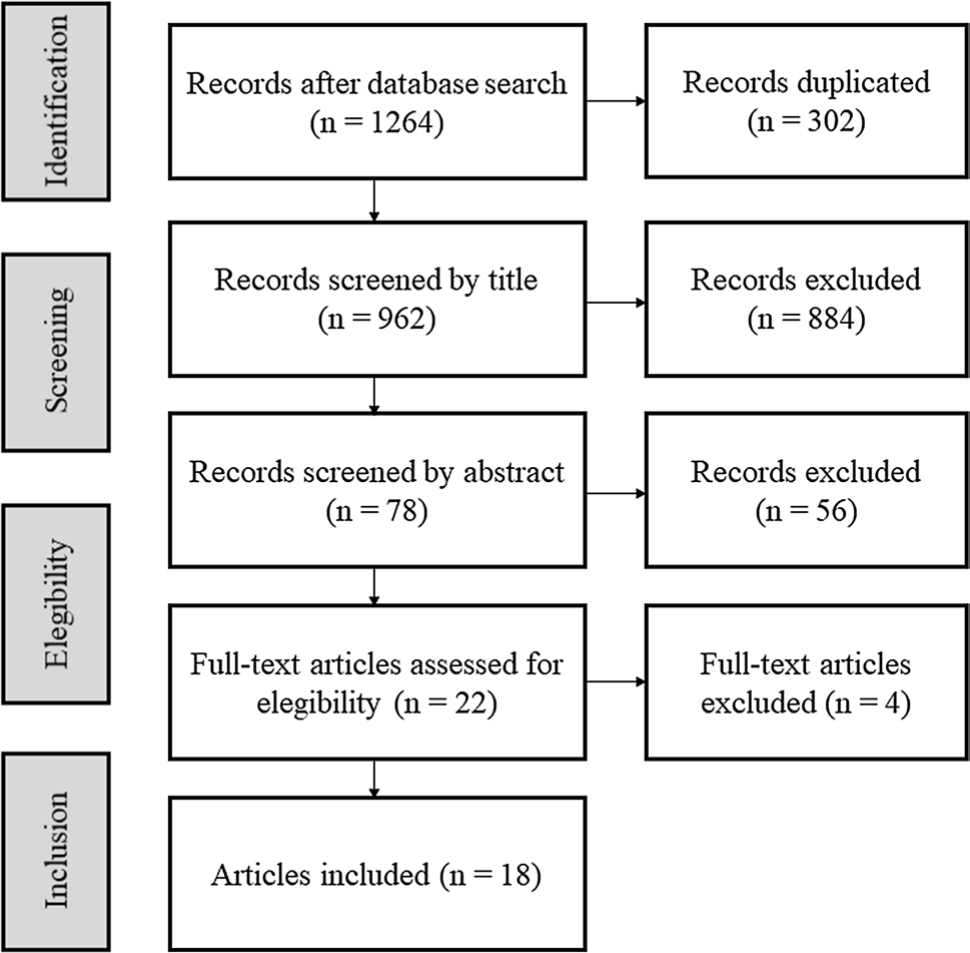
Flowchart of the selection process

Two independent reviewers selected the studies. All the studies were stored in the Mendeley desktop reference manager (Elsevier, Amsterdam, The Netherlands). The titles and abstracts of each study were read after removing any duplicates. Finally, the full articles of the abstracts identified as potentially relevant during the first screening were obtained and assessed independently for eligibility. A third reviewer took part to resolve any disagreements. The study selection process is represented in Fig. 1.

### Data Extraction

The data extracted from each study included the authors, year of publication, sample characteristics (total participants, age, sex, and health status), study design, intercourse position, and physical demands assessed during sexual intercourse. The physical demands were understood to be the physiological and kinematic capacities required to perform sexual intercourse for a specific amount of time. The physiological demands included the internal response in terms of heart rate (beats per minute, bpm) blood pressure (mmHg), rate of perceived exertion, or energy requirements (i.e., the total amount of energy expended during the activity in kilocalories, METs, which represent the ratio of the energy expended during the activity to the rate of energy expended at rest, or milliliters per kilogram per minute of oxygen, which represent the VO_2_) (Garber et al., 2011). Specifically, the VO_2_ is a variable representing the ability of the respiratory and circulatory systems to supply oxygen during any sustained physical activity (Hawkins et al., 2007). In contrast, the kinematic variables, which were selected in line with previous studies (Charbonnier et al., 2014; Lädermann et al., 2018; Sidorkewicz & McGill, 2014, 2015), included those related to the movement patterns (e.g., range of motion in degrees or penetration cycle speed) used during sexual intercourse. Once the data were extracted, a systematic qualitative review and interpretation of results was carried out.

### Methodological Quality Assessment

We know of no standard scale for assessing the methodological quality of observational research in the field of sexual intercourse. However, each study was rated based on the Effective Public Health Practice Project (EPHPP) scale, which has been used as a standard tool for assessing methodological quality in previous reviews aimed at promoting physical activity and healthy lifestyles (Norris et al., 2015; Pang et al., 2017). This scale is based on the rating (1: weak; 2: moderate; 3: strong) of six components: selection bias, study design, confounders, blinding, data collection method, and withdrawals/dropouts (Jackson & Waters, 2005). The final rating of each study may be strong (no weak ratings), moderate (one weak rating), or weak (two or more weak ratings). Based on the EPHPP guidelines, if there was a discrepancy in ratings between the reviewers, they discussed oversight (strong was the final decision), differences in criteria interpretation (moderate was the final decision), and differences in study interpretation (weak was the final decision).

## Results

### Study Selection

A total of 1264 studies were identified using the search strategy but, of these, only 962 were selected after removing duplicates. The titles were screened, and then, 78 abstracts were read. From these, 22 studies were considered for full-text reading. Four studies did not meet the inclusion criteria and, thus, a final total of 18 studies were included in this systematic review (Fig. 1).

### Characteristics of Included Studies

The characteristics of the included studies can be found in the Appendix. A total of 349 participants (men = 264; women = 85) were involved. There were 13 studies on healthy participants (*n* = 190; men = 111; women = 79) while 5 studies analyzed participants with cardiovascular diseases (*n* = 159; men = 153; women = 6). Also, 7 studies reported that the participants belonged to permanent/married couples. One study confirmed that the participants were paid. Regarding the year of publication, this spanned from 1956 to 2020 (1956–2000, *n* = 10; 2000–2020, *n* = 8).

With respect to the variables of analysis, the physical demands were examined in terms of the physiological response (*n* = 14) and the kinematics (*n* = 4). However, not all the studies collected data during real intercourse (real, *n* = 16; simulated, *n* = 2). Of the 18 studies included, 8 of them tried to control the position. In addition, the methodological quality was considered to be moderate in 7 studies and weak in 11. None of the studies were considered to be of high quality.

### Physical Demands Examined Through the Energetic System’s Physiological Response

Table 1 shows the descriptive statistics of the studies analyzing the physiological response during sexual intercourse. In terms of energy expenditure by healthy participants, the studies reported results variously as kcal, METs, and VO_2_ reached during the activity. For example, one study found that sexual intercourse expended an average of ~ 130 kcal (Zavorsky et al., 2019). Another study found that this energy expenditure was greater in men (~ 101 kcal or ~ 6 METs) than in women (~ 69.10 kcal or ~ 5.60 METs) (Frappier et al., 2013).

**Table 1:** Descriptive statistics of studies analyzing the physiological response during sexual intercourse

Reference	Variables	Results	Conclusion
Zavorsky et al. (2019)	Energy expenditure	130.00 ± 44.00 kcal	Experiencing sexual intercourse with orgasm the night before physical exercise did not significantly affect physical exercise performance on the testing day
	Mean heart rate	103.00 ± 21.00 bpm	
	Peak heart rate	~ 145 bpm	
	Duration	~ 13 min	
Frappier et al. (2013)	Absolute energy expenditure	84.80 ± 43.50 kcal (all), 101.00 ± 52.00 kcal (men) and 69.10 ± 25.60 kcal (women)	Energy expenditure during sexual intercourse was approximately 85 kcal or 3.6 kcal/min and seems to be performed at moderate intensity in young healthy adults. Therefore, sexual intercourse could potentially be considered (at times) as significant physical exercise
	Relative energy expenditure	3.60 ± 1.30 kcal/min (all), 4.20 ± 1.30 kcal/min (men) and 3.10 ± 1.00 kcal/min (women)	
	Perceived energy expenditure	87.40 ± 54.20 kcal (all), 100.00 ± 63.00 kcal (men) and 76.20 ± 43.30 kcal (women)	
	METs	5.80 ± 1.30 METs (all), 6.00 ± 1.30 METs (men) and 5.60 ± 1.20 METs (women)	
	Duration	24.70 ± 12.70 min	
Xue-ri et al. (2008)	Systolic blood pressure at different phases	Onset of excitement: 124.86 ± 13.62 mmHg (men) and 114.00 ± 17.01 mmHg (women) Onset of plateau: 141.41 ± 17.13 mmHg (men) and 121.67 ± 16.61 mmHg (women) Onset of orgasm: 128.18 ± 16.78 mmHg (men) and 121.15 ± 14.91 mmHg (women)	Blood pressure and heart rate increased slightly for a short time and recovered to the baseline level soon after sexual activity in healthy adults. Physical exhaustion during sexual activity was within the daily-life workload range
	Diastolic blood pressure at different phases	Onset of excitement: 75.23 ± 7.89 mmHg (men) and 69.52 ± 12.82 mmHg (women) Onset of plateau: 91.05 ± 13.69 mmHg (men) and 77.37 ± 15.03 mmHg (women) Onset of orgasm: 80.27 ± 10.92 mmHg (men) and 71.93 ± 14.14 mmHg (women)	
	Mean heart rate at different phases	Onset of excitement: 79.82 ± 11.75 bpm (men) and 76.89 ± 7.64 bpm (women) Onset of plateau: 91.09 ± 10.54 bpm (men) and 82.44 ± 8.06 bpm (women) Onset of orgasm: 96.36 ± 11.96 bpm (men) and 90.19 ± 10.38 bpm (women)	
Palmeri et al. (2007)	Peak heart rate	113.00 ± 24.00 bpm (men) and 105.00 ± 18.00 bpm (women)	Sexual activity is a modest physical stress compared to the first (women) and second (men) stage of the standard multistage Bruce treadmill protocol. An individual’s maximum heart rate and blood pressure during sexual activity is ~ 75% of the demands reached in maximum treadmill stress testing
	Systolic blood pressure	152.00 ± 22.00 mmHg (men) and 136.00 ± 22.00 mmHg (women)	
	RPE (1–5)	2.70 ± 0.90 (men) and 2.50 ± 0.70 (women)	
	Duration	32.38 ± 17.24 min	
Bohlen et al. (1984)	Mean heart rate at different phases	Foreplay with woman on top: 72.00 ± 10.00 bpm Stimulation with woman on top: 87.00 ± 17.00 bpm Orgasm with woman on top: 110.00 ± 24.00 bpm Foreplay with man on top: 70.00 ± 13.00 bpm Stimulation with man on top: 98.00 ± 16.00 bpm Orgasm with man on top: 127.00 ± 23.00 bpm	Significant increases in heart rate and VO_2_ above the resting baseline were observed during the foreplay phase. A modest physical effort was observed for relatively short spans, these being the maximum values achieved during the orgasm phase
	VO_2_ at different phases	Foreplay with woman on top: 5.10 ± 0.80 mL/min/kg Simulation and orgasm with woman on top: 8.70 ± 3.20 mL/min/kg Foreplay with man on top: 5.00 ± 1.00 mL/min/kg Stimulation and orgasm with man on top: 11.70 ± 3.80 mL/min/kg	
	Duration	Stimulation and orgasm with woman on top: 6.13 ± 2.90 min Stimulation and orgasm with man on top: 5.70 ± 4.35 min	
Nemec et al. (1976)	Mean heart rate	Man on top: 114.00 ± 14.00 bpm Woman on top: 117.00 ± 14.00 bpm	No differences were found in heart rate or blood pressure between sexual intercourse positions
	Systolic blood pressure	Man on top: 163.00 ± 11.00 mmHg Woman on top: 161.00 ± 18.00 mmHg	
	Diastolic blood pressure	Man on top: 81.00 ± 17.00 mmHg Woman on top: 77.00 ± 12.00 mmHg	
Littler et al. (1974)	Mean heart rate	96.32 ± 23.13 bpm	Blood pressure and heart rate increased during sexual intercourse, achieving peak values at the time of orgasm
	Systolic blood pressure	135.81 ± 28.41 mmHg	
	Diastolic blood pressure	87.71 ± 20.96 mmHg	
	Duration	13.43 ± 5.26 min	
Fox and Fox (1969)	Peak systolic blood pressure	~ 175 mmHg (man) and ~ 140 mmHg (woman)	A significant increase in systolic blood pressure was observed in the orgasm phase. This increase might be explained by the muscular effort involved or the autonomic response to the orgasm
	Duration	~ 9 min	
Bartlett (1956)	Peak heart rate	~ 170 bpm (men and women)	Peak heart rate during sexual intercourse was similar in men and women
Drory et al. (1996)	Mean heart rate	118.00 ± 21.00 bpm	Sex-related arrhythmias are likely to occur as a result of physical and emotional stress
Mann et al. (1982)	Peak heart rate	~ 131 bpm (men) and ~ 139 bpm (women)	Sexual intercourse elicited changes in the cardiovascular system. These increases in heart rate and blood pressure might be explained by isometric and dynamic exercises, breath-holding episodes, or psychological stress
	Peak systolic blood pressure	~ 237 mmHg (men) and ~ 216 mmHg (women)	
	Peak diastolic blood pressure	~ 138 mmHg (men) and ~ 127 mmHg (women)	
Stein (1977)	Peak heart rate (before and after training period)	Experimental group before training period: ~ 127 bpm Experimental group after training period: ~ 120 bpm Control group before training period: ~ 128 bpm Control group after training period: ~ 128 bpm	The exercise program, which increased aerobic capacity, may improve sexual function during sexual intercourse in patients with angina by decreasing the peak heart rate
Hellerstein and Friedman (1970)	Peak heart rate	Orgasm: 117.40 ± 4.20 bpm	The physiological demands of sexual intercourse were modest. For example, the equivalent oxygen cost was like climbing a flight of stairs, walking briskly, or performing daily-life activities
	Mean heart rate	1-min before orgasm: 101.20 ± 4.89 bpm	
	Oxygen uptake	60% VO_2MAX_ equivalent to peak heart rate: 16.00 ± 0.82 mL/min//kg 45% VO_2MAX_ equivalent to the average heart rate: 11.90 ± 0.76 mL/min/kg	
	Duration	16.30 ± 1.66 min	
Johnston et al. (1979)	Peak heart rate	Post-myocardial infarction: ~ 107.80 bpm Post-myocardial revascularization: ~ 117.80 bpm	Post-myocardial revascularization patients achieved greater peak heart rate than post-myocardial infarction patients. Sexual intercourse may lead to cardiac electrical events which are not elicited by other stimuli, so special considerations are necessary for people dealing with this disease

*bpm*, beats per minute; *VO2*, oxygen uptake; *VO2MAX*, maximal oxygen uptake; *mmHg*, millimeters of mercury; *METs*, metabolic equivalent units; *RPE*: rate of perceived exertion

Studies that analyzed the energetic demands based on oxygen uptake (VO_2_) (Bohlen et al., 1984; Hellerstein, 1970) gave results for men only. The man-on-top position elicited the greatest demands (11.70 ± 3.80 mL/min/kg), compared to the woman on top (8.70 ± 3.20 mL/min/kg), in healthy male participants (Bohlen et al., 1984). However, the VO_2_ ranged from ~ 12 to ~ 16 mL/min//kg in participants with coronary artery disease (Hellerstein, 1970).

### Physical Demands Examined Through the Heart Rate Physiological Response

When it came to healthy participants, the peak heart rates during sexual intercourse ranged from ~ 145 bpm (Zavorsky et al., 2019) to ~ 170 bpm (Bartlett, 1956). There were two studies that analyzed the heart rate pattern during the activity and both agreed that the heart rate increases progressively from foreplay to orgasm, with an average in men of between ~ 96 bpm (Xue-Rui et al., 2008) and ~ 127 bpm (Bohlen et al., 1984). In addition, differences were found between men and women (men: ~ 96 bpm; women: 90 bpm) (Xue-Rui et al., 2008). Although the man-on-top position elicited greater heart rate demands (~ 127 bpm) in men than the woman on top (~ 110 bpm) (Bohlen et al., 1984), another study found these demands to be higher for the woman-on-top position (~ 117 bpm) than the man-on-top position (~ 114 bpm) (Nemec et al., 1976).

The greatest heart rate reached by participants with hypertension was ~ 121 bpm (men) and ~ 139 bpm (women) (Mann et al., 1982), which was achieved during the orgasm phase (Hellerstein, 1970). Furthermore, heart rates during sexual intercourse in these patients may decrease by following an exercise program (from ~ 127 bpm to ~ 120 bpm) (Stein, 1977).

### Physical Demands Examined Through the Blood Pressure Physiological Response

The research studies that collected blood pressure data showed that systolic blood pressure was greater in healthy men than in healthy women (Fox & Fox, 1969; Nemec et al., 1976; Palmeri et al., 2007; Xue-Rui et al., 2008). The same results were also found when analyzing diastolic blood pressure (Nemec et al., 1976; Xue-Rui et al., 2008). In addition, the pattern of changes in blood pressure during sexual intercourse showed differences in the onset of the plateau in peak blood pressure values for men (systolic: ~ 141.41 mmHg; diastolic: 91.05 mmHg) and women (systolic: ~ 121.67 mmHg; diastolic: 77.37 mmHg) (Xue-Rui et al., 2008).

One study collected data from patients with hypertension (Mann et al., 1982). The difference between men (systolic: ~ 237 mmHg; diastolic: 138 mmHg) and women (systolic: ~ 216 mmHg; diastolic: ~ 127 mmHg) was also observed in these participants.

### Physical Demands Examined Through the Perceived Exertion Response

The perceived exertion response was analyzed in healthy men and women (Frappier et al., 2013; Palmeri et al., 2007). The rate of perceived exertion, which was measured using a five-point scale, was greater in men (~ 2.70) than women (~ 2.50) (Palmeri et al., 2007). In addition, the perceived energy expenditure was similar to the absolute energy expenditure in both men (~ 100 kcal) and women (~ 76.20 kcal) (Frappier et al., 2013).

### Physical Demands Examined Through the Duration of Sexual Intercourse

A total of 8 studies reported the average duration of sexual intercourse in their participants (Bohlen et al., 1984; Fox & Fox, 1969; Frappier et al., 2013; Hellerstein, 1970; Johnston & Fletcher, 1979; Littler et al., 1974; Palmeri et al., 2007; Zavorsky et al., 2019). While many variables account for the sexual intercourse duration (e.g., the data collection protocol, positions, psychological artifacts, etc.), the greatest mean durations were ~ 32.38 min in healthy participants (Palmeri et al., 2007) and ~ 18.60 min in patients with cardiovascular disease (Johnston & Fletcher, 1979).

### Physical Demands Examined Through the Kinematics

Table 2 shows the descriptive statistics for the kinematics of sexual intercourse. A total of three studies analyzed the kinematics of sexual intercourse in different positions in healthy adults (Charbonnier et al., 2014; Sidorkewicz & McGill, 2014, 2015). The movements were cyclic and the greatest flexion demands on the lumbar spine were found in the missionary positions (missionary 1: ~ 10.70°; missionary 2: ~ 20.50°) for women (Sidorkewicz & McGill, 2015), whereas the missionary 1 (~ 16.76°) and side-lying (~ 30.97°) positions elicited the greatest lumbar flexion movement in men (Sidorkewicz & McGill, 2014).

**Table 2:** Descriptive statistics of studies analyzing the kinematics of sexual intercourse

Reference	Variables	Results	Conclusion
Lädermann et al. (2018)	Minimal subacromial space height (mm)	Scorpion: 0.91 ± 1.06 mm Missionary: 1.27 ± 0.03 mm Superman: 1.76 ± 1.60 mm Watering can: 6.10 ± 0.22 mm Basset hound: 6.20 ± 0.17 mm	Scorpion, missionary and superman may be stressful positions for the rotator cuff since these positions showed less subacromial space height than the watering can and basset hound positions
Sidorkewicz and McGill (2014)	Mean lumbar spine angular range of motion	Quadruped 1: 0.60 ± 11.30° Quadruped 2: 7.50 ± 11.50° Missionary 1: 10.70 ± 4.80° Missionary 2: 20.50 ± 7.40° Side-lying: 3.60 ± 7.60°	The movements were cyclic and performed in the sagittal plane of motion. The flexion demands on the lumbar spine that occurred during the missionary 1 and 2 positions lead to the conclusion that these positions are least recommended for the flexion-intolerant patient. Since quadruped 2 and side-lying positions lead to spine motion within the extension, these may be recommended for the flexion-intolerant patient. However, quadruped 2 and side-lying could be contraindicated for the extension-intolerant patient
	Percentage of maximum sagittal range of motion	Quadruped 1: 35.50 ± 3.50% Quadruped 2: 33.50 ± 3.50% Side-lying: 26.20 ± 3.50% Missionary 1: 22.40 ± 4.80% Missionary 2: 18.30 ± 3.80%	
Sidorkewicz and McGill (2014)	Mean lumbar spine angular range of motion	Quadruped 1: 7.14 ± 9.84° Quadruped 2: 8.21 ± 10.00° Missionary 1: 9.35 ± 14.69° Missionary 2: 16.76 ± 12.42° Side-lying: 30.97 ± 13.54	The movements were mainly performed in the sagittal plane of motion. None of the participants got to 100% of their spine range of motion at any position. In addition, side-lying and missionary 2 are not positions recommended for flexion-intolerant patients given the hip flexion requirements of these positions. Quadruped 1, quadruped 2, and missionary 1 (in this order) would be recommended instead
	Penetration cycle speed	Quadruped 1: 1.80 ± 0.72 cycles/s Quadruped 2: 1.81 ± 0.72 cycles/s Missionary 1: 1.37 ± 0.68 cycles/s Missionary 2: 1.33 ± 0.49 cycles/s Side-lying: 1.50 ± 0.60 cycles/s	
Charbonnier et al. (2014)	Hip flexion–extension range of motion	Position 1: 1.00 ± 2.80° (man) and 5.00 ± 3.50° (woman) Position 2: 82.00 ± 1.50° (man) and 74.00 ± 2.10° (woman) Position 3: 37.00 ± 5.70° (man) and 96.00 ± 3.50° (woman) Position 4: 30.00 ± 2.10° (man) and 22.00 ± 2.80° (woman) Position 5: 39.00 ± 5.40° (man) and 108.00 ± 5.70° (woman) Position 6: 23.00 ± 5.70° (man) and 100.00 ± 3.50° (woman) Position 7: 17.00 ± 1.40° (man) and 56.00 ± 3.50° (woman) Position 8: 76.00 ± 5.80° (man) and 82.00 ± 2.40° (woman) Position 9: 22.00 ± 6.40° (man) and 45.00 ± 4.20° (woman) Position 10: 10.00 ± 2.10° (man) and 95.00 ± 6.40° (woman) Position 11: 3.00 ± 0.70° (man) and 49.00 ± 2.10° (woman) Position 12: 44.00 ± 5.70° (man) and 70.00 ± 4.20° (woman)	Sexual intercourse positions required flexion, abduction and mainly external rotation in woman. Specifically, the most demanding positions in flexion (> 95°) were position 3, 5, 6, and 10 while the most demanding positions in abduction were position 4, 7, 10, and 12 (> 32°). However, external rotation was dominant for all male positions, with external rotation values greater than 40° in position 5, 6, 8, and 11
	Hip abduction–adduction range of motion	Position 1: 1.00 ± 11.30° (man) and 16.00 ± 0.70° (woman) Position 2: 32.00 ± 2.30° (man) and 14.00 ± 1.60° (woman) Position 3: 8.00 ± 10.60° (man) and 27.00 ± 2.10° (woman) Position 4: 4.00 ± 16.30° (man) and 35.00 ± 6.40° (woman) Position 5: 18.00 ± 3.20° (man) and 6.00 ± 17.70° (woman) Position 6: 15.00 ± 4.90° (man) and 26.00 ± 0.70° (woman) Position 7: 5.00 ± 9.90° (man) and 34.00 ± 2.80° (woman) Position 8: 21.00 ± 5.20° (man) and 29.00 ± 2.50° (woman) Position 9: 1.00 ± 5.70° (man) and 7.00 ± 2.80° (woman) Position 10: 2.00 ± 9.90° (man) and 35.00 ± 2.10° (woman) Position 11: 1.00 ± 9.90° (man) and 26.00 ± 1.40° (woman) Position 12: 2.00 ± 4.90° (man) and 32.00 ± 1.40° (woman)	
	Hip external–internal rotation range of motion	Position 1: 34.00 ± 7.80° (man) and 18.00 ± 4.20° (woman) Position 2: 34.00 ± 4.60° (man) and 16.00 ± 3.90° (woman) Position 3: 14.00 ± 8.50° (man) and 2.00 ± 0.70° (woman) Position 4: 6.00 ± 7.80° (man) and 20.00 ± 1.40° (woman) Position 5: 41.00 ± 4.50° (man) and 9.00 ± 2.10° (woman) Position 6: 40.00 ± 3.50° (man) and 7.00 ± 1.40° (woman) Position 7: 30.00 ± 7.10° (man) and 4.00 ± 6.40° (woman) Position 8: 47.00 ± 3.70° (man) and 9.00 ± 4.20° (woman) Position 9: 4.00 ± 9.20° (man) and 9.00 ± 1.40° (woman) Position 10: 18.00 ± 4.20° (man) and 4.00 ± 4.20° (woman) Position 11: 42.00 ± 17.00° (man) and 17.00 ± 9.90° (woman) Position 12: 11.00 ± 14.10° (man) and 15.00 ± 2.10° (woman)	

*mm*, millimeters, °, degrees; cycles/s, penetration cycles per second. A description of the positions is available in Table 3

A further study analyzed the hip range of motion in 12 positions (Table 3) with two participants (Charbonnier et al., 2014). Sexual intercourse positions required hip flexion, abduction, and mainly external rotation in the woman (Charbonnier et al., 2014). Specifically, the most demanding positions for the woman in terms of flexion (> 95°) were positions 3, 5, 6, and 10 while the most demanding positions in terms of abduction were positions 4, 7, 10, and 12 (> 32°) (Charbonnier et al., 2014). In contrast, for the man, hip external rotation was predominant for all the positions, with external rotation values greater than 40° in positions 5, 6, 8, and 11 (Charbonnier et al., 2014).

**Table 3:** Description of sexual intercourse positions

Reference	Description
Lädermann et al. (2018)	**Scorpion**: the female stays in a quadruped position and the male, who is in a front plank position, enters from behind **Missionary**: the female lies on her back with the knees flexed and the male on top with his face opposite hers **Superman**: the male holds the female face down. Specifically, his arms are wrapped around the hips while he stands up with knees flexed and enters from behind **Watering can**: the male lies on his back with the knees slightly flexed and the female on top with her face opposite his. Also, the female’s knees are brought up to his side and her arms are wrapped around the back of his neck **Basset hound**: the female stays in a quadruped position with the trunk parallel to the bed and the male enters from behind while on his knees
Sidorkewicz and McGill (2014)	**Quadruped 1**: the female stays in a quadruped position with the trunk flexed and the male enters from behind while on his knees **Quadruped 2**: the female stays in a quadruped position with the trunk parallel to the bed and the male enters from behind while on his knees **Missionary 1**: the female lies on her back with the knees slightly extended and the male on top with his face opposite hers **Missionary 2**: the female lies on her back with the knees flexed and the male on top with his face opposite hers **Side-lying**: the couple lies on the right or left side but facing the same direction while the male enters from behind
Sidorkewicz and McGill (2014)	**Quadruped 1**: the female stays in a quadruped position with the trunk flexed and the male enters from behind while on his knees **Quadruped 2**: the female stays in a quadruped position with the trunk parallel to the bed and the male enters from behind while on his knees **Missionary 1**: the female lies on her back with the knees slightly extended and the male on top with his face opposite hers **Missionary 2**: the female lies on her back with the knees flexed and the male on top with his face opposite hers **Side-lying**: the couple lies on the right or left side but facing the same direction while the male enters from behind
Charbonnier et al. (2014)	**Position 1**: the female lies on her chest with the knees extended and the male on top with a similar position and entering from behind **Position 2**: this is a variation of “Position 1” in which the female flexed the right hip so that her right leg is between the male’s legs **Position 3**: the female stays in a quadruped position with the trunk parallel to the bed and the male enters from behind while on his knees **Position 4**: the female lies on her back with the knees flexed and the male on top with his face opposite hers. However, the male lays his hands on the bed with elbows extended so that there is a distance of separation between his and her chests **Position 5**: the male lies on his left side and the female is positioned in a perpendicular direction to the male. Specifically, the female lies on her back with the knees and hips flexed, which are held by the male **Position 6**: the female lies on her back with the knees and hips flexed (i.e., legs up) and the male on top with his face opposite hers **Position 7**: the female lies on her back with the knees flexed and the male on top with his face opposite hers **Position 8**: this is similar to the side-lying position, but the male lies on his left side and the female lies on her right side with her face opposite his **Position 9**: both partners stand up and the male enters from behind while the female has her arms supported on a nearby surface **Position 10**: the male lies on his back with the knees slightly flexed and the female on top with her face opposite his. Also, the female’s knees are brought up to his side and her trunk is leant forward **Position 11**: the male lies on his back with the knees slightly flexed and the female on top with her face opposite his. Also, the female’s knees are brought up to his side and her trunk is perpendicular to the male **Position 12**: the male sits on a chair while he enters the female (who is sitting on him) from behind

Another variable of analysis was the speed of the penetration cycle in men, which varied based on the sexual intercourse position (Sidorkewicz & McGill, 2014). Specifically, the greatest values were observed in quadruped variants (quadruped 1: ~ 1.80 cycles per second; quadruped 2: ~ 1.81 cycles per second), followed by side-lying (~ 1.50 cycles per second) and the missionary positions (missionary 1: ~ 1.37 cycles per second; missionary 2: ~ 1.33 cycles per second) (Sidorkewicz & McGill, 2014).

Lastly, there was one study that collected data on the kinematic demands on the rotator cuff, based on the height of the subacromial space (Lädermann et al., 2018). This study found that the subacromial space height was lower for the watering can (~ 6.10 mm) and basset hound (~ 6.20 mm) positions compared to the scorpion (~ 0.91 mm), missionary (~ 1.27 mm), or superman (~ 1.76 mm) positions (Lädermann et al., 2018).

## Discussion

This is the first systematic review investigating the physical demands of sexual intercourse. It included a total of 18 studies. The main findings were that all the studies analyzed either the physiological demands or the kinematics of sexual intercourse, but no studies combined both analyses. Sexual intercourse can elicit an energy expenditure of ~ 100 kcal (or ~ 6 METs) per activity, mean heart rates between ~ 90 and ~ 130 bpm, and peak heart rates up to ~ 170 bpm. Therefore, sexual intercourse may be a significant contributor to physical health, and it can be considered as a form of physical activity. However, these physical demands may vary depending on health status, intercourse position, activity duration, intercourse phase, lubrication, erections, and sex differences. Furthermore, although little information is available concerning the kinematics of sexual intercourse, it is suggested that some positions (e.g., the cowgirl, scorpion, missionary, or superman) may not be recommended for individuals with low back pain, hip pathologies, or shoulder injuries. More high-quality studies are required to gain a better understanding of the physical demands of this activity.

### Characteristics of Included Studies

These studies analyzed kinematic and physiological parameters independently. Based on the definition of physical activity as being any musculoskeletal movement that results in energy expenditure (Caspersen et al., 1985), the possibility of making conclusions may be limited—for instance, in terms of energy expenditure during sexual intercourse—without knowing the kinematic parameters such as range of motion, speed of movement, or the specific energetic cost of getting and maintaining an erection, orgasm, and ejaculation. In consequence, sex tracking technologies (e.g., smart condoms or wearable sensors synchronized with physiological monitors) may be a productive alternative for future research on this topic (Kreitmair, 2018; Oliva-Lozano et al., 2020).

In addition, there are significant concerns regarding the context for testing the physical demands of sexual intercourse. For example, some studies collected data in laboratory settings. However, this suggests that psychological artifacts may have been introduced into the natural intercourse situation, even if only minimally, and therefore such artifacts cannot be absolutely eliminated (Fox & Fox, 1969). Moreover, the data were collected both from real and simulated intercourse. Although participants were encouraged to move as naturally as possible during simulated intercourse, there was no actual intercourse, and this is its main limitation. Nonetheless, these data also have their own limitations—many studies did not mention or control what type of sexual activity the participants engaged in, requiring only that it culminated in intercourse and male orgasm (Palmeri et al., 2007). Moreover, some participants got paid (Bohlen et al., 1984)—a recent study considered that paying participants to have sex as part of a scientific study might be controversial (Zavorsky et al., 2019).

In addition, there were no studies of high quality, and this limits the generalization of the results, which are not amenable to meta-analysis because there is a potentially high risk of bias. Only 6 studies have been published within the last 10 years, which is difficult to explain given current technological advances. However, this might be interpreted in various ways. Perhaps in recent decades, sexologists have become wiser about all the dependent variables that exist, and have come to recognize how difficult it is to carry out properly controlled, high-quality research on the demands of sexual intercourse. Although we do not assume that newer studies would be better, this systematic review highlights the lack of high-quality studies, which hopefully will be published in future.

### Physical Demands Examined Through the Energetic System’s Physiological Response

Two studies reported that sexual intercourse elicited an energy expenditure of between 100 and 130 kcal in healthy adults (Frappier et al., 2013; Zavorsky et al., 2019). In addition, the mean intensity was ~ 6 METs (Frappier et al., 2013), which can be categorized as moderate intensity physical activity (Garber et al., 2011; Haskell et al., 2007). Examples of activities performed at this intensity include jogging (speed: 6 km/h), swimming leisurely (not lap swimming), or stationary rowing (Ainsworth et al., 2011). However, these studies did not analyze the energetic cost of different positions.

One research study analyzed energetic demands based on VO_2_ in different positions during the activity (Bohlen et al., 1984). VO_2_ is a measure used when reporting data on energetic demands and it is considered more accurate than absolute measures (e.g., METs or kcal) (Ainsworth et al., 1993; Garber et al., 2011). However, only one study reported the energetic requirements in participants with cardiovascular disease, with the VO_2_ ranging from ~ 12 to ~ 16 mL/min//kg (Hellerstein, 1970), which suggests that they also performed the activity at moderate intensity (Ainsworth et al., 1993; Garber et al., 2011; Haskell et al., 2007). Nonetheless, these studies did not analyze the energetic requirements for different positions during sexual intercourse in unhealthy participants. Future research could investigate the effect of different positions on energy expenditure during sexual intercourse in order to estimate which are the most or least demanding for men and women. Moreover, these studies might use perceived effort questionnaires and dispense with collecting any energy expenditure data in the laboratory as previous researchers have (Frappier et al., 2013).

### Physical Demands Examined Through the Heart Rate Physiological Response

Healthy participants reached peak heart rates between ~ 145 bpm (Zavorsky et al., 2019) and ~ 170 bpm (Bartlett, 1956). Nevertheless, it is not known how much of that is related to hip thrusts, the orgasmic experience, erection, or ejaculation. Within the context of sexual intercourse, it is important to note that this heart rate response is not only a consequence of the activity’s physical demands but also depends on emotional components, especially those characterized by the orgasmic experience (Bohlen et al., 1984; Falk, 2001; Xue-Rui et al., 2008). This may explain why the heart rate progressively increases from the foreplay to the orgasm phase (Bohlen et al., 1984; Xue-Rui et al., 2008). However, differences were observed between the heart rate response in men and in women (men: ~ 96 bpm; women: 90 bpm) (Xue-Rui et al., 2008). This might be because men are the more active partner in sexual intercourse when in specific positions (e.g., the missionary or the man-on-top) (Bohlen et al., 1984; Levin, 2007). Nonetheless, understanding the heart rate response to any physical activity is not all about sex differences. Indeed, it is estimated that both sedentary and trained women exhibit greater maximum heart rate (bpm) than men. However, it is important to mention that the heart rate response may vary depending on other factors such as age or cardiorespiratory fitness (Whyte et al., 2008).

In contrast to the above findings, the heart rate reached by participants with cardiovascular diseases was ~ 139 bpm in women and ~ 121 bpm in men (Mann et al., 1982). Although the data on women’s sexual physiology are limited, these differences might be explained by the facts that a marked increase in heart rate accompanying the orgasm may be observed (Bartlett, 1956; Xue-Rui et al., 2008) and that women may experience multiple orgasms within the same period of sexual intercourse (Gérard et al., 2021). However, women do not reach orgasm from penetration alone and these differences may be a result of sympathetic contributions to arousal in women (Lorenz et al., 2012; Meston, 2000).

Another interesting finding of this systematic review was that following a regular exercise program for 16 weeks decreased the mean heart rate (from ~ 127 bpm to ~ 120 bpm) during sexual intercourse in patients with cardiovascular diseases (Stein, 1977). Given that regular exercise may have such a protective effect for these patients, physicians could advise patients to participate in these programs before engaging in sexual activities (Kumagai et al., 2020; Stanton et al., 2018; Stein, 1977).

### Physical Demands Examined Through the Blood Pressure Physiological Response

All the studies observed that systolic blood pressure (Fox & Fox, 1969; Nemec et al., 1976; Palmeri et al., 2007; Xue-Rui et al., 2008) and diastolic (Nemec et al., 1976; Xue-Rui et al., 2008) blood pressure were greater in healthy men than in healthy women. This difference was also observed in patients with cardiovascular diseases, but with higher blood pressure values in both men and women (Mann et al., 1982). In this regard, it is worth mentioning that sex differences play a significant role in regulating blood pressure (e.g., men typically have higher blood pressure than women at similar ages) (Maranon & Reckelhoff, 2013; Reckelhoff, 2001). Furthermore, although blood pressure increases in other physical activities (Hedberg et al., 2009; Laukkanen & Kurl, 2012), what stands out about sexual intercourse is how the isometric and dynamic positions (e.g., men in the missionary positions or women in the quadruped positions) increase blood pressure (Mann et al., 1982).

### Physical Demands Examined Through the Perceived Exertion Response

Even though there were only two studies measuring the perceived exertion of the participants during sexual intercourse, both agreed that sexual intercourse elicited moderate-intensity (Garber et al., 2011) physical demands (Frappier et al., 2013; Palmeri et al., 2007). These studies used self-designed questionnaires to measure perceived effort (Frappier et al., 2013; Palmeri et al., 2007). This suggests that future research should apply valid and reliable tools since criterion-related validity and reliability represent the empirical relationship between perceived effort and physiological measures, which best describe exercise intensity (Chen et al., 2002). Indeed, specific tools need to be designed for the sexual intercourse context because the strength of the relationship between perceived effort and physiological response may vary based on the characteristics of the activity, which implies that a lot of contextual variables need to be considered (Chen et al., 2002; Garber et al., 2011).

### Physical Demands Examined Through the Duration of the Sexual Intercourse

When considering the studies that analyzed the duration of sexual intercourse (Table 1), the results suggest that the sexual intercourse duration is highly dependent on the couple themselves; this may be related, among other factors, to the age of the participants (e.g., duration of foreplay, experimental environment), since a negative correlation was found between age and the sexual intercourse duration (Palmeri et al., 2007). It was also observed that the duration of the activity was not always controlled. For instance, researchers from a previous study mentioned that they did not control what type of sexual activity the participants engaged in as long as they acknowledged it concluded with sexual intercourse and male orgasm (Palmeri et al., 2007). From a physical activity perspective, the duration of the exercise does not necessarily imply greater health benefits (Garber et al., 2011); rather, it is the intensity that greatly determines the energetic demands (i.e., the amount of energy required to perform any activity) and, therefore, the possible health outcomes (Garber et al., 2011).

### Physical Demands Examined Through the Kinematics

The scarcity of studies on the kinematics of sexual intercourse may be explained by the difficulties associated with doing high-quality research in this area. Some positions might not be recommended for individuals with low back pain as they have a restricted range of motion in the lumbar spine (i.e., flexion-intolerant or extension-intolerant) (Sidorkewicz & McGill, 2014, 2015), perhaps due to the combination of kinematic position and kinetic force on the lumbar spine (Saraceni et al., 2020). For example, there are positions that may demand high lumbar spine flexion for men (e.g., side-lying) and women (e.g., missionary) (Sidorkewicz & McGill, 2014, 2015). However, a previous systematic review reported that using parameters other than range of motion, such as angular velocity or acceleration, was recommended by the included studies when assessing low back patients (Papi et al., 2018). Given the current developments in wearable sensors, these could be used in future studies to collect such information during sexual intercourse (Ciuti et al., 2014; Oliva-Lozano et al., 2020).

Only one study collected data on the pace of the intercourse based on the penetration cycle speed (Sidorkewicz & McGill, 2014). These results suggest that positions such as the quadruped, which elicited greater speed, and therefore greater intensity, may increase the energy required (i.e., kcal, METs, or VO_2_) (Garber et al., 2011). However, as the speed increases, the risk of hip dislocation may increase as well (Charbonnier et al., 2014; Dahm et al., 2004; Harmsen et al., 2018).

To the best of the authors’ knowledge, scant information is available concerning hip range of motion (Charbonnier et al., 2014). The main limitation was that the data were collected from a limited sample size (one man and one woman); nonetheless, the sexual intercourse simulation required high levels of hip flexion and abduction, with mainly external rotation in the woman, whereas the man was exposed to less hip range of motion but pronounced external rotation (Charbonnier et al., 2014). Consequently, certain positions might not be recommended for people (particularly women) suffering from hip pathologies (e.g., hip arthritis, total hip arthroplasty surgery) (Charbonnier et al., 2014) because the risk of total hip arthroplasty dislocation exists (Dahm et al., 2004). Based on the results from a survey conducted by hip surgeons, whose aim was to report a set of specified guidelines for resuming safe sexual intercourse after total hip arthroplasty, 51 of the surgeons reported at least one case of patients with total hip arthroplasty dislocation during sexual intercourse (Dahm et al., 2004). Also, it was observed that 43.5% of the patients, who expected to be sexually active after surgery, perceived this expectation as unfulfilled (Harmsen et al., 2018).

In this regard, a recent study concluded that intercourse positions such as the scorpion, missionary, or superman may cause rotator cuff degeneration because of friction on the undersurface of the acromion (Lädermann et al., 2018). This is due to the reduced or minimal subacromial space height (Lädermann et al., 2018). However, it is not only subacromial impingement that might lead to rotator cuff degeneration but also the scapular morphology (Moor et al., 2014).

### Study Limitations

The present systematic review has its own limitations, the main one being that scant methodologically solid research has been carried out on the physical demands existing during sexual intercourse; this weakens the interpretation of the results obtained to date and the potential of this review. For example, future studies of the kinematics of sexual intercourse need to consider many more parameters than just range of motion or penetration cycle. This is of interest if we know all the variables associated with greater (or lesser) range of motion or thrusting sequence timing. To obtain such data, far greater sample sizes are required that consider a wider range of variables. However, this objective may be easily stymied by the implicit biases involved in who volunteers as subjects for such studies. There are also contextual variables, like the size and body mass of the couple, the penis dimension, the penis angle during erections, the amounts of lubrication, or the couple’s dyadic quality, that were not controlled in previous studies but might affect the demands of sexual intercourse. Moreover, most of the studies mainly analyzed the physical demands on men, while little is known about the physical demands on women. Furthermore, the lack of diversity in the literature should be recognized given that scant data exist on sexual activity in same-sex couples or non-penetrative sexual activity. Consequently, future studies should consider all the variables that previous authors failed to define or qualify.

### Conclusion

This is the first systematic literature review on physical demands during sexual activity. It comprised a total of 18 studies, all of which analyzed either the physiological demands or kinematics of sexual intercourse, although none of the studies combined both analyses. Sexual intercourse can elicit moderate physical demands, which may vary depending on the participants’ health status, the intercourse position, the activity’s duration, the intercourse phase, and the sex of the participant. However, there are contextual variables (e.g., cardiorespiratory fitness, orgasmic experience, amounts of lubrication, or the couple’s dyadic quality) that are difficult to control but might influence the demands of sexual intercourse. Furthermore, only four studies, which were conducted on healthy adults, analyzed the kinematics of sexual intercourse in different positions. The movement pattern was cyclic, with the greatest flexion demands on the lumbar spine in women being observed in the missionary positions; in contrast, for men, the missionary 1 and side-lying positions elicited the greatest lumbar flexion movement. Regarding hip joint movement during sexual intercourse, flexion, abduction, and mainly external rotation characterized the women’s movement, while external rotation characterized the hip joint movement in men. Therefore, some intercourse positions may increase the risk of low back pain and hip or shoulder injury. Consequently, a kinematic analysis of pelvic rotation in the sagittal plane would be helpful. Although the lumbar flexion in specific intercourse positions (e.g., missionary) may help rotate the woman’s pubis in a way that increases physical contact between the clitoris and the penis, sexologists might wonder if the rate of orgasm for women in these positions relates to insufficient pelvis rotation for achieving adequate clitoral stimulation.

## Appendix

See Table

**Table 4:** Characteristics of the included studies

Reference	N	Sex	Health state	Age (years)	Study design	Position	Instruments	Variables	Level of evidence
Zavorsky et al. (2019)	8	7 men and 1 woman	Healthy	28.50 ± 5.00	Cross-sectional study in a private, unmonitored setting (real intercourse)	Uncontrolled	Heart rate monitor RS-200 (Polar Electro, Kempele, Finland)	Physiological	Moderate
Lädermann et al. (2018)	2	1 man and 1 woman	Healthy	35.00 ± 5.65	Cross-sectional study in laboratory settings (simulated intercourse)	Missionary; Scorpion; Watering can; Basset hound; Superman	MRI system 1.5 T HDxT system (General Electric Healthcare, Milwaukee, USA) 24 optoelectronic motion capture cameras Vicon MXT50S (Vicon Motion Systems, Oxford, UK)	Kinematic	Weak
Sidorkewicz and McGill (2014)	10	Women	Healthy	29.80 ± 8.00	Cross-sectional study in laboratory settings (real intercourse: 20 s for each position)	Missionary 1; Missionary 2; Quadruped 1; Quadruped 2; Side-lying	Electromagnetic motion capture system 3SPACE Isotrak (Polhemus, Vermont, USA)	Kinematic	Moderate
Sidorkewicz and McGill (2014)	10	Men	Healthy	29.30 ± 6.90	Cross-sectional study in laboratory settings (real intercourse: 32 s for each position)	Missionary 1; Missionary 2; Quadruped 1; Quadruped 2; Side-lying	8 optoelectronic motion capture cameras Vicon MX20 + (Vicon Motion Systems, Oxford, UK)	Kinematic	Weak
Charbonnier et al. (2014)	2	1 man and 1 woman	Healthy	28.50 ± 3.53	Cross-sectional study in laboratory settings (simulated intercourse)	12 positions	24 optoelectronic motion capture cameras Vicon MXT40S (Oxford Metrics, Oxford, UK)	Kinematic	Weak
Frappier et al. (2013)	42	21 men and 21 women (permanent couples)	Healthy	22.60 ± 2.80	Longitudinal study at home (real intercourse: 1 session per week for 1 month)	Uncontrolled	SenseWear armband (Bodymedia, Pittsburgh, USA); Perceived exertion questionnaire	Physiological	Moderate
Xue-ri et al. (2008)	49	27 women and 22 men	Healthy	40.45 ± 7.80	Cross-sectional study at home (real intercourse)	Man on top	Ambulatory blood pressure monitor (Meditech, Budapest, Hungary); Dynamic electrocardiogram DCG (Meditech, Budapest, Hungary)	Physiological	Moderate
Palmeri et al. (2007)	32	19 men and 13 women	Healthy	53.00 ± 7.50	Cross-sectional study at home (real intercourse)	Uncontrolled	Ambulatory blood pressure monitor 90,217 (SpaceLabs Medical, Issaquah, USA); Heart rate monitor S610 (Polar Electro, Kempele, Finland); Perceived exertion questionnaire	Physiological	Moderate
Bohlen et al. (1984)	10	Men (married), who were paid	Healthy	33.20	Longitudinal study (2 sessions of real intercourse) in an experimental room	Man on top; Woman on top	Ambulatory ECG; Expired air through computerized lab system, polarographic O_2_ gas analyzer, and an infrared CO_2_ gas analyzer	Physiological	Weak
Nemec et al. (1976)	10	Men (married)	Healthy	29.30	Longitudinal study (4 sessions of real intercourse over 1-week period) at home	Man on top; Woman on top	Ambulatory ECG Model 350 (Holter Electrocardiocorder) and ambulatory blood pressure monitor, Model 1260 (Roche, Cranbury, USA)	Physiological	Weak
Littler et al. (1974)	7	6 men and 1 woman	Healthy	28.14 ± 5.58	Cross-sectional study at home (real intercourse)	Uncontrolled	Grandjean Arteriocath 3 (Plastimed B.P., Saint-Leu-la-Forêt, France); Air Shields ECG system	Physiological	Weak
Fox and Fox (1969)	2	1 man and 1 woman (married)	Healthy	35.50 ± 3.53	Longitudinal study (4 sessions of real intercourse) in the habitual environment of the couple’s own bedroom	Man on top	Winston blood pressure follower (Dynamics Corp. Of America, Greenwich, USA)	Physiological	Weak
Bartlett (1956)	6	3 men and 3 women (married)	Healthy	22–30	Cross-sectional study in an experimental room (real intercourse)	Uncontrolled	Electrocardiograph	Physiological	Weak
Drory et al. (1996)	88	Men (permanent / married couples)	Patients with coronary artery disease	52	Cross-sectional study (real intercourse)	Uncontrolled	Ambulatory electrocardiograph Siemens Siretape System (Siemens, Henkestrasse, Germany)	Physiological	Weak
Mann et al. (1982)	11	8 men and 3 women	Patients with hypertension	42.09 ± 8.89	Cross-sectional study (real intercourse)	Uncontrolled	Brachial artery catheter, Medilog (Oxford Medical Systems, Clearwater, USA)	Physiological	Weak
Stein (1977)	22	Men	Post-myocardial infarction patients	46–54	2 sessions from a clinical trial study (real intercourse)	Uncontrolled	Ambulatory Holter Electrocardiocorder	Physiological	Moderate
Hellerstein and Friedman (1970)	14	Men (married)	Patients with coronary artery disease	47.50	Cross-sectional study at home (real intercourse)	Uncontrolled	Ambulatory Holter electromagnetic tape-recorder and bicycle ergometer	Physiological	Weak
Johnston et al. (1979)	24	21 men and 3 women	Post-myocardial infarction and revascularization patients	49.60 ± 1.69	Cross-sectional study (real intercourse)	Uncontrolled	Ambulatory electrocardiocorder	Physiological	Moderate

4.
